# Convalescent Plasma for Pregnant Women with COVID-19: A Systematic Literature Review

**DOI:** 10.3390/v13071194

**Published:** 2021-06-22

**Authors:** Massimo Franchini, Federico Prefumo, Gianpaolo Grisolia, Valentino Bergamini, Claudia Glingani, Marlene Pisello, Francesca Presti, Marco Zaffanello

**Affiliations:** 1 Department of Hematology and Transfusion Medicine, Carlo Poma Hospital, I-46100 Mantova, Italy; massimo.franchini@asst-mantova.it (M.F.); claudia.glingani@asst-mantova.it (C.G.); 2 Department of Clinical and Experimental Sciences, University of Brescia, I-25123 Brescia, Italy; federico.prefumo@unibs.it; 3 Obstetrics and Gynecology Unit, Carlo Poma Hospital, I-46100 Mantova, Italy; gianpaolo.grisolia@asst-mantova.it (G.G.); marlene.pisello@asst-mantova.it (M.P.); 4 Department of Obstetrics and Gynecology, Azienda Ospedaliera Universitaria Integrata, I-37126 Verona, Italy; valentino.bergamini@aovr.veneto.it (V.B.); francesca.presti@aovr.veneto.it (F.P.); 5 Department of Surgical Sciences, Dentistry, Gynecology and Pediatrics, University of Verona, I-37126 Verona, Italy

**Keywords:** convalescent plasma, COVID-19, fetus, newborn, hyperimmune plasma, pregnancy, SARS-CoV-2

## Abstract

The treatment of COVID-19 is particularly critical in pregnant women, considering the potential teratogenic effects of antiviral agents and the immune-depression related with pregnancy. The aim of this review is to systematically examine the current evidence on the clinical use of convalescent plasma during pregnancy. The electronic databases Medline PubMed Advanced Search Builder, Scopus, Web Of Science and Google Scholar were searched (until 1 January 2021). Inclusion criteria were pregnant women with COVID-19 (or SARS-CoV-2 infection), in whom convalescent plasma (or hyperimmune plasma) was used as treatment. We searched clinical trial registries (censored 5 January 2021) for eligible studies under way. After elimination of duplications, the initial search yielded 79 potentially relevant records, of which 67 were subsequently excluded. The 12 remaining records were case reports involving 12 pregnancies. Six of the mothers were reported to be well, two were reported to have preeclampsia, and in one case each the maternal outcome was described as survival, clinical improvement, discharged with oxygen and rehabilitation. With regard to the neonates, two were declared to be well, four had transient morbidity, two were critically ill and one died; normal ongoing pregnancies, but no post-delivery information, were reported for the remaining three cases. Clinical trials under way or planned to investigate the use of convalescent plasma for COVID-19 during pregnancy are lacking. This is the first systematic review of the literature regarding the treatment of COVID-19 in pregnancy. The published literature data seem to indicate that convalescent plasma administered to pregnant women with severe COVID-19 provides benefits for both the mother and the fetus. The quality of the available studies is, however, very limited since they are all case reports and thus suffer from relevant reporting bias.

## 1. Introduction

At the beginning of 2020 a pandemic caused by a novel coronavirus began to have devastating effects worldwide. The quickly identified etiological agent was named severe acute respiratory syndrome coronavirus 2 (SARS-CoV-2) because of its similarity to the coronavirus which caused the SARS epidemic in 2002. At the time of writing, nearly 109 million people have been infected by the virus and more than 2.4 million people have died of the disease it causes, coronavirus disease 2019 (COVID-19). From 20 to 26 January 2021 the world recorded 101,366 deaths, or an average of 14,000 every day [1]. It has been clear since the beginning of the pandemic that severe forms of the disease could have dramatic effects even in young patients without comorbidities, including pregnant women [2,3]. Pneumonia, which is the main complication of COVID-19, is the most important non-obstetric infection in pregnancy, being a significant cause of maternal and neonatal morbidity and mortality, particularly when associated with other comorbid conditions, including obesity, cardiovascular disorders and respiratory diseases [4]. A quarter of the cases of pneumonia in pregnancy need intensive care treatment with mechanical ventilation and may be complicated by premature rupture of the membranes, fetal growth restriction, preterm labor and intrauterine or neonatal death [5,6,7]. A number of studies have therefore examined the potential adverse maternal, fetal and neonatal outcomes of COVID-19 during pregnancy [8,9,10,11,12]. Even though there is currently no evidence of an increased risk of miscarriage in relation to COVID-19, the infection is reported to be associated with a relatively high rate of preterm birth, pre-eclampsia, Cesarean section and perinatal death [8].

An intense debate has started on therapeutic possibilities for COVID-19 in pregnancy. The management of the disease is essentially driven by the symptoms. Asymptomatic or pauci-symptomatic women do not require inpatient care or medications, but simply need monitoring of respiratory function for up to 2 weeks for evidence of deterioration. Antiviral treatments such as remdesivir and drugs such as hydroxychloroquine and azithromycin have been administered, but their use is currently not recommended [13]. Plasma from individuals who have recovered from COVID-19, a blood component that is not contraindicated in pregnant women and that has been transfused safely for at least 30 years, deserves special consideration. Following numerous observations of its efficacy in patients with severe SARS-CoV-2 infection, convalescent plasma (CP) has also been tried in critically ill pregnant women with COVID-19.

The aim of this paper is to systematically review the current evidence on the clinical use of CP during pregnancy.

## 2. Sources

The electronic databases Medline PubMed Advanced Search Builder, Scopus, Web Of Science and Google Scholar were searched (until 1 February 2021) using the following medical subject heading (MeSH) terms and text words (their combinations and truncated synonyms): [HYPERIMMUNE PLASMA or CONVALESCENT PLASMA] and [PREGNANT OR PREGNANCY] and [COVID-19 or SARS-COV-2]. Duplicate reports were removed and the abstracts of the articles retrieved were screened. The full texts were then analyzed and the reference lists were examined for further articles missed in the primary search. The search strategy had no limitations for language, geographical area or race. Inclusion criteria were all original studies that described pregnant women with COVID-19 (or SARS-COV-2 infection) in whom CP (or hyperimmune plasma) was used as a treatment. Exclusion criteria were reviews of published studies, studies published only as abstracts, letters or conference proceedings, discussion papers, animal studies or editorials.

Initial screening of titles identified potentially relevant studies; this was followed by screening of abstracts and then review of the full texts. All titles and abstracts were evaluated independently by two reviewers (M.Z., M.F.), not blinded to the authors’ names or journal of publication. Any initial disagreements were resolved by consensus unless the two review authors could not reach an agreement, in which case a third author (F.P.) was consulted to make a decision. No ethical approval was required for this study.

## 3. Selection of Studies

Two independent reviewers (M.Z., M.F.) evaluated the articles potentially meeting the inclusion criteria and retrieved the full texts. Studies that did not fulfil all inclusion criteria were excluded; reasons for exclusion are reported.

When data from the same cohort were presented in more than one article, only the reports that most directly evaluated therapy with CP (or hyperimmune plasma) and COVID-19 (or SARS-COV-2 infection) in pregnancy were included in this review. Full texts were screened, and bibliographic details, as well as data regarding study design, participants, disease severity, interventions and outcomes were recorded in predefined forms. All data, numerical calculations and graphic extrapolations were independently confirmed. The methodological quality of the studies was assessed with the tool described by Murad et al. [14] which identifies eight items related to selection (*n* = 1), ascertainment (*n* = 2), causality (*n* = 4) and reporting (*n* = 1).

Due to the lack of study homogeneity, a narrative synthesis of the results is provided. The study was registered with the Prospective Registering of Systematic Reviews (PROSPERO) database (registration number: CRD42021237705).

## 4. Ongoing Trials Involving Pregnant Patients

We searched clinical trial registries (censored 5 January 2021) for eligible studies under way or planned to investigate the use of CP for COVID-19 during pregnancy. The six online databases used for this research were https://clinicaltrials.gov/; https://eudract.ema.europa.eu/; https://www.clinicaltrialsregister.eu/; https://www.who.int/ictrp/network/en/; http://www.chictr.org.cn/abouten.aspx and https://www.irct.ir/.

## 5. Results

**Figure 1:**
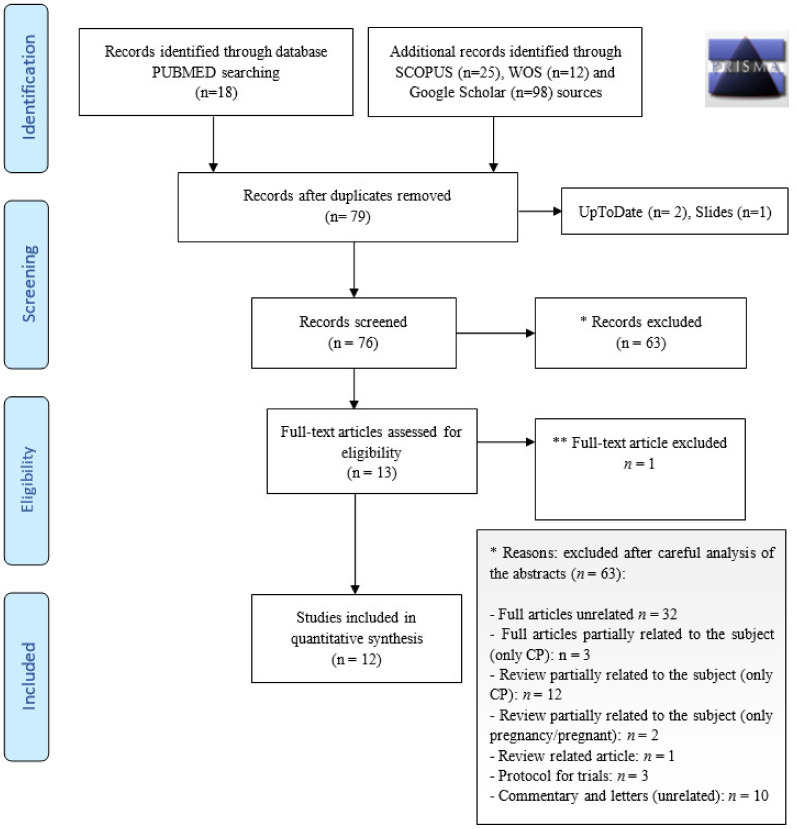
PRISMA 2009 Flow Diagram. * Records were excluded because: although full articles, they were unrelated to the subject; they were only partially related to the subject (i.e., they mentioned only pregnancy or only convalescent plasma treatment); they were not full articles, (i.e., they were reviews, review-related articles, trial protocols, commentaries or letters. ** One article was removed after careful analysis of the full paper.

After screening for duplicates, the initial search yielded 79 records as detailed in the PRISMA flow diagram (Figure 1).

The articles identified were all in English. At this step, three records were excluded because they were not published in scientific journals. Further screening of abstracts led to the exclusion of another 63 records because they were full articles unrelated to the subject (*n* = 32), full articles partially related to the subject (only CP treatment, no pregnant women; *n* = 3), reviews partially related to the subject (only pregnant women or only CP treatment: *n* = 14), a review-related article (*n* = 1), protocols for trials (all unrelated; *n* = 3), commentaries or letters (*n* = 10).

At that point, 13 articles were assessed for eligibility [15,16,17,18,19,20,21,22,23,24,25,26,27]. After reading the full texts, one article was removed: Chong et al. [15] reported the case of a 41-year-old woman, 32 weeks pregnant, who developed severe acute respiratory distress syndrome (ARDS) from SARS-CoV-2 infection, necessitating emergency Cesarean section in the intensive care room. The baby was delivered and, being critically ill, was intubated for 48 h. The woman’s condition postoperatively was unstable and she was successfully treated with CP given over the following 24 h (i.e., after delivery of her child) [15].

At the end of the assessment phase, 12 records were included in this review; all were case reports involving a total of 12 pregnancies [16,17,18,19,20,21,22,23,24,25,26,27]. Table 1 presents the characteristics of the studies included in the final review.

Table 2 shows the quality of the studies based on the assessment of four domains: selection (maximum score 1), ascertainment (maximum score 2), causality (maximum score 4) and reporting (maximum score 1).

According to this assessment, the overall quality of the studies included in this systematic review was low. The reports originated from the USA (*n* = 5) [19,20,24,26,27], Italy (*n* = 2) [17,22], Iran (*n* = 2) [16,18], Mexico [23], Qatar [21] and China [25]. The age of the women ranged between 22 [20] and 42 years [27].

Clinical conditions before CP treatment were severe ARDS in 11 women and mild ARDS in one [17]. No comorbidity was declared in five cases [18,22,23,25,27]. Two women had one comorbid condition (obesity) [16,17] and five had multiple comorbid conditions [19,20,21,24,26]. The gestational age at CP treatment ranged between 21 + 2 [16] and 36 + 2 [24] weeks. All patients received other medications during their stay in hospital: antivirals of the nucleotide analogue class (remdesivir, *n* = 5), protease inhibitors (lopinavir/ritonavir; *n* = 4), steroids (*n* = 8), heparin (*n* = 7), hydroxychloroquine (*n* = 5) and humanized monoclonal antibodies (tocilizumab, *n* = 2). Additional maternal interventions were described in nine patients, of whom six were given invasive mechanical ventilation, three received extracorporeal membrane oxygenation [21,25,27] and three required tracheostomy [20,22,27].

The neutralizing antibody titer in the CP was reported in only one case [17]. The absence of CP-related adverse events was explicitly stated in eight cases [16,17,19,22,23,25,26,27], while the other four case reports made no mention of adverse events after the procedure [18,20,21,24]. In three reports, the pregnancy was described as ongoing after CP treatment [16,22,26]. In eight cases Caesarean section was performed (at gestational age range 25–36 weeks) [18,19,20,21,22,24,25,27] while two babies were delivered vaginally [17,23]. In one report, the patient was discharged with regular outpatient visits [16].

The mother was reported to be well at discharge in six cases; to have preeclampsia in two cases and there was one case each of maternal survival, clinical improvement, discharged with oxygen and rehabilitation. In two cases the neonate was declared to be well [17,18], while there were four reports of transient morbidity [21,23,24,27], two cases of postnatal intensive care admission [19,20] and one death [19]. The other three reports described normal ongoing pregnancies, but without information on the post-natal outcome [16,22,26]. Since these were stated to be normal ongoing pregnancies a good neonatal outcome is likely.

The searches of clinical trial registries for eligible studies under way or planned to investigate the use of CP for COVID-19 during pregnancy were negative. The three protocols for a randomized controlled trial we found during our online search of databases [28,29,30] excluded pregnant women from the design.

This report is the first systematic review of the literature regarding the use of CP to treat COVID-19 in pregnancy. The analysis of the published literature data seems to support the beneficial maternal and fetal effects of CP administration to pregnant women with severe COVID-19.

The sudden SARS-CoV-2 pandemic at the beginning of 2020 posed a major challenge to physicians because there was no specific pre-existing therapy fort this new virus. As a consequence, the therapeutic efforts were initially focused on optimizing respiratory care, managing thrombotic and inflammatory complications by using anticoagulation and corticosteroids, and repurposing existing antiviral therapies [31]. Unfortunately, almost all the initially promising agents (i.e., hydroxychloroquine, lopinavir/ritonavir and remdesivir) failed to demonstrate a beneficial effect [32,33,34]. Considering the lack of effective anti-SARS-CoV-2 drugs and the initial positive experience from China [35], the first country hit by the new coronavirus, CP, an old therapy that had been used with apparent success in many epidemics and outbreaks since the Spanish flu in 1918 [36,37,38], was proposed again also for COVID-19 [39]. Pooled analyses from the numerous trials published so far substantially confirm the beneficial effect of CP, especially when it contains high titers of neutralizing anti-SARS-CoV-2 antibodies and when used early during the clinical course of severe COVID-19 [40,41]. In addition, several case reports and case series have documented the efficacy of CP in severe COVID-19 when administered in patients with acquired and congenital immunodeficiency [42].

SAR-CoV-2 infection in pregnant women seems to have negative effects on both maternal and neonatal outcomes. Premature birth, maternal death, intrauterine fetal death and neonatal death were the most frequent complications [43,44]. The rates of maternal and neonatal death were found to be 5% and 6%, respectively [45]. The issue of treatment of COVID-19 is more critical in pregnant women, considering the potential teratogenic effect of antiviral agents and the immune-depression related to pregnancy, which could be responsible for an inadequate antibody response to the SARS-CoV-2 infection.

Pregnant women are a traditionally poorly represented group in drug studies, but a high-risk group with regard to unwanted maternal-fetal events because of the unique physiology of pregnancy with implications for fetal and neonatal drug exposure. However, given the complexity of participation in drug trials during pregnancy, many clinical drug trials exclude pregnant women [46].

Even though passive immunotherapy by means of CP transfusion is attractive in this group of patients with such particular characteristics, only 12 cases of administration of CP to pregnant women have been published so far [16,17,18,19,20,21,22,23,24,25,26,27]. While advanced maternal age did not seem to represent a particular risk for severe COVID-19 complications, the median age of the cases at presentation being 32.0 years (range, 22–42 years), the majority of cases were recorded during the third trimester (median gestational age: 27.9 weeks, range 22–36 weeks). This latter finding is in accordance with literature data indicating that the third trimester of pregnancy is the most vulnerable period for severe SARS-CoV-2 infection [11]. In all reported cases the CP was administered to critically ill patients with moderate/severe ARDS. The severity of the respiratory disease was confirmed by the high rate (7/12, 58.3%) of invasive procedures required (invasive mechanical ventilation, tracheostomy, extracorporeal membrane oxygenation) to improve life-threatening hypoxia.

Seven (58%) of the 12 pregnant women had at least one co-morbidity (mostly obesity, diabetes and asthma) documenting that, as previously reported, pregnant women positive for SARS-CoV-2 with co-morbidities are more likely to develop complications than those without [47,48]. In the majority of cases (56%, 6/9), two CP units were required to obtain a clinical improvement. The transfusion of the first CP unit was performed at a median of 2 days (range, 1–17 days) after admission to hospital. Early infusion (within 72 h of hospital admission) is important to obtain the best anti-viral effect of hyperimmune plasma, as documented by a recent randomized controlled trial [49]. Unfortunately, the anti-SARS-CoV-2 neutralizing titer, an important parameter for evaluating CP efficacy, was available for only two of the CP units. Notably, no adverse effects of CP transfusion were recorded, confirming the safety of this treatment [50]. Hyperimmune plasma was used in combination or as second-line treatment following the failure of several other drugs, including antibiotics, steroids, anticoagulants (low molecular weight heparin), hydroxychloroquine and antiviral agents (lopinavir/ritonavir, remdesivir). The maternal outcome was positive in all the reported cases. Cesarean section was required in the majority of pregnancies (7/12, 58%), in accordance with literature data on the mode of delivery during pregnancy-associated severe COVID-19 [10,11].

Strength: This is the first systematic review of the literature regarding the treatment of COVID-19 with CP in pregnancy. There is a lack of available, specifically designed scientific research on the safety of CP for pregnant women and fetuses.

Limitations: The main limitations and biases of the present study are that it includes only clinical case reports. Another bias (reporting bias) is that cases with a positive outcome are preferentially reported, possibly precluding representativeness of the whole population of pregnant women treated with CP.

## 6. Conclusions

The analysis of the published literature data seems to support the beneficial maternal and fetal effects of CP administered to pregnant women with severe COVID-19. The quality of the available studies is, however, low since they are only case reports and thus suffer from relevant reporting bias. The understanding of the role of CP in the treatment of pregnancy-associated COVID-19 could be improved by data from registries and adequately powered, specifically designed trials also enrolling pregnant women.

## Figures and Tables

**Table 1:** Summary of the cases of COVID-19 in pregnant women treated with convalescent plasma.

Author, Year [Ref]	Design	Country	Age, y	Gestational Age	Severity of Disease	Comorbidity	Procedures	CP Treatment				Other Medications	Outcome	
								Units Transfused	NAbT	Days from Hospitalization	AR		Maternal	Fetal/Neonatal
Grisolia, 2020 [17]	CR	Italy	29	24 w and 2 d	Mild ARDS	Class I obesity	VD	2	160	+1, +4	None	Ceftriaxone, azithromycin, hydroxychloroquine, methylprednisolone, LMWH	Maternal well-being	Full-term, well neonate with VD
Zhang, 2020 [25]	CR	China	31	35 w and 2 d	Severe ARDS	-	CD (35 w), IMV, ECMO	1	NR	+17	None	Lopinavir/ritonavir, ribavirin, imipenem, vancomycin	Maternal survival	Neonatal death due to intrauterine asphyxia
Anderson, 2020 [26]	CR	USA	35	22 w and 2 d	Severe ARDS	Type 2 DM, asthma, class III obesity	Forego delivery (25 w)	1	NR	+1	None	Remdesivir, ceftriaxone, azithromycin, hydroxychloroquine, hydrocortisone, LMWH	Maternal well-being	Normal ongoing pregnancy
Donzelli, 2020 [22]	CR	Italy	34	27 w and 4 d	Severe ARDS	-	IMV, PP, tracheostomy, CD (30 w)	2	NR	+2, +3	None	Clarithromycin, ceftriaxone, betamethasone, LMWH	Maternal well-being	Normal ongoing pregnancy
Jacobson, 2021 [27]	CR	USA	42	26 w	Severe ARDS	-	CD (29 w), IMV, PP, ECMO, tracheostomy	1	NR	+2	None	Remdesivir, dexamethasone, azithromycin, ceftriaxone	Discharged with home oxygen	Neonatal adrenal insufficiency, then good condition
Magallanes-Garza, 2020 [23]	CR	Mexico	33	27 w and 4 d	Severe ARDS	-	VD (39 w), IMV	2	NR	+4, +5	None	Lopinavir/ritonavir, LMWH, azithromycin, ceftaroline, methylprednisolone	Maternal well-being	Neonatal GR
Pelayo, 2020 [24]	CR	USA	35	36 w and 2 d	Severe ARDS, PE	Asthma, class III obesity, ileal carcinoma, HCV	IMV, CD (36 w)	1	NR	NR	NR	Methylprednisolone, remdesivir, heparin, vancomycin, ceftriaxone	Discharged to acute inpatient rehabilitation unit	Neonate intubation due to hypoxia, then positive outcome
Jafari, 2020 [18]	CR	Iran	26	36 w and 1 d	Moderate ARDS	-	CD (36 w)	NR	NR	NR	NR	Favipiravir, meropenem, azithromycin, hydroxychloroquine	Maternal well-being	Neonate well
Easterlin, 2020 [20]	CR	USA	22	23 w and 6 d	Severe ARDS	Tuberous sclerosis, nephrectomy, leiomyosarcoma	CD (25 w), PP, tracheostomy	NR	NR	NR	NR	Azithromycin, hydroxychloroquine, remdesivir, tocilizumab, LMWH	Pre-eclampsia, post-delivery critically ill condition	Critically ill preterm neonate with severe respiratory failure
Soleimani, 2020 [16]	CR	Iran	30	21 w and 2 d	Severe ARDS	Class II obesity	-	2	NR	+10, +11	None	Lopinavir/ritonavir, LMWH, azithromycin, methylprednisolone	Maternal well-being	Normal ongoing pregnancy
Lam, 2020 [19]	CR	USA	30	23 w and 1 d	Severe ARDS	Type 2 DM, hypertension, pre-eclampsia	CD (25 w)	NR	NR	+1	None	Remdesivir, dexamethasone, azithromycin, ceftriaxone	Pre-eclampsia, discharged on day +28	Neonate intubated due to hypoxia, stable condition
Yaqoub, 2020 [21]	CR	Qatar	33	32 w	Severe ARDS	Asthma, gestational diabetes	CD (32 w), IMV, ECMO	2	NR	+5	NR	Lopinavir/ritonavir, tocilizumab, hydroxychloroquine, azithromycin, ceftriaxone	Clinical improvement, discharged on day +40	Neonate intubated due to hypoxia, then positive outcome

Abbreviations: AR, adverse reactions to CP infusion; ARDS, acute respiratory distress syndrome; CD, Cesarean delivery; CR, case report; d, days; DM, diabetes mellitus; ECMO, extracorporeal membrane oxygenation; GR, growth restriction; IMV, invasive mechanical ventilation; LMWH, low-molecular weight heparin; NAbT, neutralizing antibody titer; NR, not reported; PE, pulmonary embolism; PP, prone positioning; VD, vaginal delivery; y, years; w, weeks.

**Table 2:** Quality assessment of the included studies.

Author	Selection	Ascertainment	Causality	Reporting
Grisolia, 2020 [17]	★	★	★★	★
Zhang, 2020 [25]	★	★★	★	★
Anderson, 2020 [26]	★	★	★★	★
Donzelli, 2020 [22]	★	★★	★★	★
Jacobson, 2021 [27]	★	★★	★	★
Magallanes-Garza, 2020 [23]	★	★★	★★	★
Pelayo, 2020 [24]	★	★	★	★
Jafari, 2020 [18]	★	★	★	★
Easterlin, 2020 [20]	★	★	★	★
Soleimani,2020 [16]	★	★	★★	★
Lam, 2020 [19]	★	★	★	★
Yaqoub, 2020 [21]	★	★★	★	★

★ represent scoring system.

## Data Availability

Not applicable.
